# Causal associations between insulin and Lp(a) levels in Caucasian population: a Mendelian randomization study

**DOI:** 10.1186/s12933-024-02389-7

**Published:** 2024-08-29

**Authors:** Mateusz Lejawa, Marcin Goławski, Martyna Fronczek, Tadeusz Osadnik, Francesco Paneni, Massimiliano Ruscica, Natalia Pawlas, Małgorzata Lisik, Maciej Banach

**Affiliations:** 1 Department of Pharmacology, Faculty of Medical Sciences in Zabrze, Medical University of Silesia, Katowice, Poland; 2 Department of Cardiology, University Heart Center, University Hospital Zurich, Rämistrasse 100, 8091 Zurich, Switzerland; 3 Department of Cardiology, Center for Translational and Experimental Cardiology (CTEC), Zurich University Hospital, University of Zurich, Wagistrasse 12, 8952 Schlieren, Switzerland; 4 Department of Cardio-Thoracic-Vascular Diseases, Foundation IRCCS Cà Granda Ospedale Maggiore Policlinico, Milan, Italy; 5 Department of Pharmacological and Biomolecular Sciences “Rodolfo Paoletti”, Università degli Studi di Milano, Milan, Italy; 6 Outpatient Clinic, Maria Sklodowska-Curie National Research Institute of Oncology Gliwice Branch, Gliwice, Poland; 7 Department of Preventive Cardiology and Lipidology, Medical University of Lodz, Lodz, Poland

**Keywords:** Lipoprotein(a), Insulin, Mendelian randomization

## Abstract

**Background:**

Numerous observational studies have demonstrated that circulating lipoprotein(a) [Lp(a)] might be inversely related to the risk of type 2 diabetes (T2D). However, recent Mendelian randomization (MR) studies do not consistently support this association. The results of in vitro research suggest that high insulin concentrations can suppress Lp(a) levels by affecting apolipoprotein(a) [apo(a)] synthesis. This study aimed to identify the relationship between genetically predicted insulin concentrations and Lp(a) levels, which may partly explain the associations between low Lp(a) levels and increased risk of T2D.

**Methods:**

Independent genetic variants strongly associated with fasting insulin levels were identified from meta-analyses of genome-wide association studies in European populations (GWASs) (*N* = 151,013). Summary level data for Lp(a) in the population of European ancestry were acquired from a GWAS in the UK Biobank (*N* = 361,194). The inverse-variance weighted (IVW) method approach was applied to perform two-sample summary-level MR. Robust methods for sensitivity analysis were utilized, such as MR‒Egger, the weighted median (WME) method, MR pleiotropy residual sum and outlier (MR-PRESSO), leave-one-out analysis, and MR Steiger.

**Results:**

Genetically predicted fasting insulin levels were negatively associated with Lp(a) levels (β = − 0.15, SE = 0.05, *P* = 0.003). The sensitivity analysis revealed that WME (β = − 0.26, SE = 0.07, *P* = 0.0002), but not MR‒Egger (β = − 0.22, SE = 0.13, *P* = 0.11), supported a causal relationship between genetically predisposed insulin levels and Lp(a).

**Conclusion:**

Our MR study provides robust evidence supporting the association between genetically predicted increased insulin concentrations and decreased concentrations of Lp(a). These findings suggest that hyperinsulinaemia, which typically accompanies T2D, can partially explain the inverse relationship between low Lp(a) concentrations and an increased risk of T2D.

**Supplementary Information:**

The online version contains supplementary material available at 10.1186/s12933-024-02389-7.

## Background

Lipoprotein(a) [Lp(a)] is classified as a highly atherogenic lipoprotein that consists of a lipid-rich domain, apolipoprotein B100, and apolipoprotein(a) [apo(a), not to be confused with apolipoprotein A] encoded by the *LPA* gene on chromosome 6 (q25.3–q26) [1]. The serum Lp(a) concentration ranges from 70 to 90%, as determined by *LPA* gene variants [2]. The variability in Lp(a) concentrations is primarily due to “copy number variations” in the number of repeats of Kringle-IV 2 (KIV-2) domains and, to a lesser extent, by single-nucleotide polymorphisms (SNPs) within and in the proximity of this gene, as well as loss-of-function mutations [3]. In addition, sex, diet, physical activity, hormonal balance, and kidney and liver diseases may influence Lp(a) levels [4, 5]. It is worth noting that in the interpretation of available results, there is a significant drawback related to the strongly skewed distribution of Lp(a) concentrations in a population and differences between patients from different ethnic groups [6].

The physiological role of Lp(a) in humans has not yet been fully clarified. However, it has been suggested that it is responsible for the transport of oxidized phospholipids and affects the processes of carcinogenesis, inflammation, coagulation, and wound healing [7]. The impact of elevated Lp(a) concentrations on the development of cardiovascular disease (CVD) has been confirmed by epidemiological studies, including genome-wide association (GWASs) and Mendelian randomization (MR) studies [8–11].

Several studies have suggested that there is an inverse association between Lp(a) concentration and the risk of type 2 diabetes (T2D), whereby people with a low Lp(a) concentration have a greater risk of T2D [12–14]. However, it is unclear whether this inverse association between Lp(a) concentration and T2D is causal. MR analysis is a method that can explore causal associations between exposures and outcomes arising from observational epidemiological studies [15]. Nevertheless, evidence from MR studies has not confirmed the causal relationship between low Lp(a) concentrations and a greater risk of T2D [16–18]. Indeed, some data indicate that not the Lp(a) concentration but the high number of KIV-2 repeats may be associated with an increased risk of T2D [19, 20]. The difficulty in interpreting the results of MR studies can be partly explained by differences in analysis strategies and the heterogeneity of instrumental variables, as individual SNPs may have different functional significance (only some are loss-of-function mutations) and differ in their relationships with KIV-2 repeats [21].

Previously published in vitro studies have shown that insulin can regulate Lp(a) synthesis in the liver [22, 23]. Specifically, increased insulin levels may reduce Lp(a) synthesis. This relationship may partially explain the association of Lp(a) with T2D observed in epidemiological studies because increasing tissue insulin resistance, leading to T2D, is accompanied by hyperinsulinaemia. Unfortunately, there is insufficient in vivo data to properly validate these results.

Several MR analyses focusing on the impact of insulin on lipid traits were conducted in the past, however, the impact of insulin on Lp(a) was not extensively studied [24, 25]. We have hypothesized that the reason for the apparent correlation of low Lp(a) with T2D without clear confirmation from MR studies may be explained by reverse causation where the increased fasting insulin affects Lp(a) levels.

This study applied a two-sample MR approach using summary-level GWAS data to examine this hypothesis to identify whether genetically predicted insulin levels influence Lp(a) concentrations in the Caucasian population.

## Materials and methods

### Study design

In this study, we used a two-sample MR approach utilising publicly available GWAS summary statistics to investigate the causal relationship between insulin and Lp(a). This approach eliminates the need for additional ethical approval or informed consent [26]. This study adhered to the Strengthening the Reporting of Observational Studies in Epidemiology (STROBE) guidelines, which are mainly designed for MR studies (STROBE-MR) (Supplementary Table 1, Additional file 1) [27].

### Exposure data

IVs (instrument variables) for BMI-adjusted fasting insulin were derived from meta-analysis of aggregated GWASs for *N* = 151,013 individuals of European ancestry without diabetes (a subset of the total number of 281,416 individuals included in the full study) [28]. Access to the GWAS summary statistics was obtained through the OpenGWAS project developed at the MRC Integrative Epidemiology Unit (IEU) [29]. The GWAS ID corresponding to insulin was “ebi-a-GCST90002238”.

### Outcome data

Summary-level data for Lp(a), Apolipoprotein B (ApoB) and LDL-cholesterol (LDL-C) from UK Biobank, which were inverse-rank normalized by the Neale laboratory (*N* = 361,194), were obtained through the OpenGwas project [29]. The GWAS IDs corresponding to Lp(a) ApoB and LDL-C are “ukb-d-30790_irnt”, “ukb-d-30640_irnt” and “ukb-d-30780_irnt” respectively. There were no overlapping populations between the GWASs exposure and outcome.

### Selection of instrumental variables

To be valid IVs in MR, SNPs must meet three core assumptions: (1) relevance, (2) independence or exchangeability and (3) exclusion restriction. Whether a SNP meets those criteria cannot be confirmed but can be falsified. Therefore, SNPs were considered appropriate IVs if they were associated with insulin levels of genome-wide significance (*p*-value < 5.00e−08) and not in linkage disequilibrium (LD) with each other (r^2^ < 0.001) within a clumping distance of 10,000 kb. For each IV, the variance (R^2^) was calculated using the following formula: R^2^ = 2×beta^2^×EAF×(1 − EAF), where EAF is the effect allele frequency and beta is the association estimate. Additionally, F-statistics were calculated based on the formula F=(beta/se)^2^, where ‘beta’ and ‘se’ refer to the genetic association of SNPs with the exposure and its standard error, respectively [15]. SNPs with F-statistics < 10 were considered weak IVs and excluded from the analysis [30]. Full lists of all SNPs at different stages of selection are presented in Supplementary Tables 2–3 (Additional file 2). To prevent potential pleiotropy, associations of the selected SNPs with confounding factors influencing Lp(a) concentration were evaluated using PhenoScanner V2 [31]. For further analysis, two sets of IVs were used: all identified SNPs except pleiotropic variants (conservative analysis) and all identified SNPs with pleiotropic variants (liberal analysis).

### Statistical analyses

The MR approach was used to estimate the association between the genetically predicted concentration of fasting insulin and Lp(a). In the first step, variant harmonization between datasets was conducted to confirm that the association between SNPs and the exposure and between SNPs and the outcome reflected the same alleles. If the selected SNPs were unavailable in the outcome dataset, they were replaced with proxy SNPs with an LD of r^2^ > 0.8 or excluded from further MR analysis. Furthermore, outlier pleiotropic variants were excluded via MR-PRESSO [32]. After that, two-sample MR analyses were performed with the inverse-variance weighted (IVW) method with multiplicative random effects as the primary analysis for evaluating the causal effect estimates in our study [33].

In the next step, sensitivity analyses were performed using the following methods: MR‒Egger, weighted median (WME), and MR pleiotropy residual sum and outlier (MR-PRESSO) [32, 34, 35]. Additionally, the presence of horizontal pleiotropy was assessed by calculating the MR‒Egger intercept deviation [36]. The Cochran Q statistic was also calculated to measure heterogeneity between variant-specific causal estimates [37]. Furthermore, to examine the possibility that reverse causality exists in these studies, the MR Steiger test was performed and a bidirectional two-sample MR analysis between genetically predicted Lp(a) and insulin was performed using the same approach described earlier [38]. Finally, leave-one-out analyses were performed to determine the possible effect of SNPs on the causal estimates. As an additional sensitivity analysis, we used variants employed as insulin IVs in Buchmann and colleagues’ paper to validate our findings on a different IVs set [39]. Moreover, due to the complex structure of Lp(a), we conducted additional MR analyses on the primary components of Lp(a), such as LDL-C and ApoB, to measure the mediating effects of insulin on each component separately. In these analyses, we used only the IVs set from the liberal analysis. A *p*-value (*P*) of < 0.05 in this study was considered to indicate statistical significance for all tests. All these analyses were conducted with R software (version 4.1.1) [40] using “TwoSampleMR” (version 0.5.6) [41] and the “MR-PRESSO” package (version 1.0) [32].

## Results

According to the criteria set, 38 IVs associated with fasting insulin levels were obtained (Supplementary Table 3, Additional file 2). After searching in PhenoScanner, 22 SNPs were removed from the conservative analysis because they are related to known or likely confounders (kidney diseases, lipid parameters, psychosocial and lifestyle outcomes) for Lp(a) level (Supplementary Table 4, Additional file 2, records with exclusion reasons for SNPs are presented in bold) to remove pleiotropic SNPs that may affect Lp(a) levels through mechanisms other than affecting apo(a) expression via insulin [42]. No outliers were detected by the MR-PRESSO. The genetic variants used in conservative, liberal, and additional sensitivity analyses are listed in Supplementary Tables 5–7 (Additional file 3). 

**Fig. 1:**
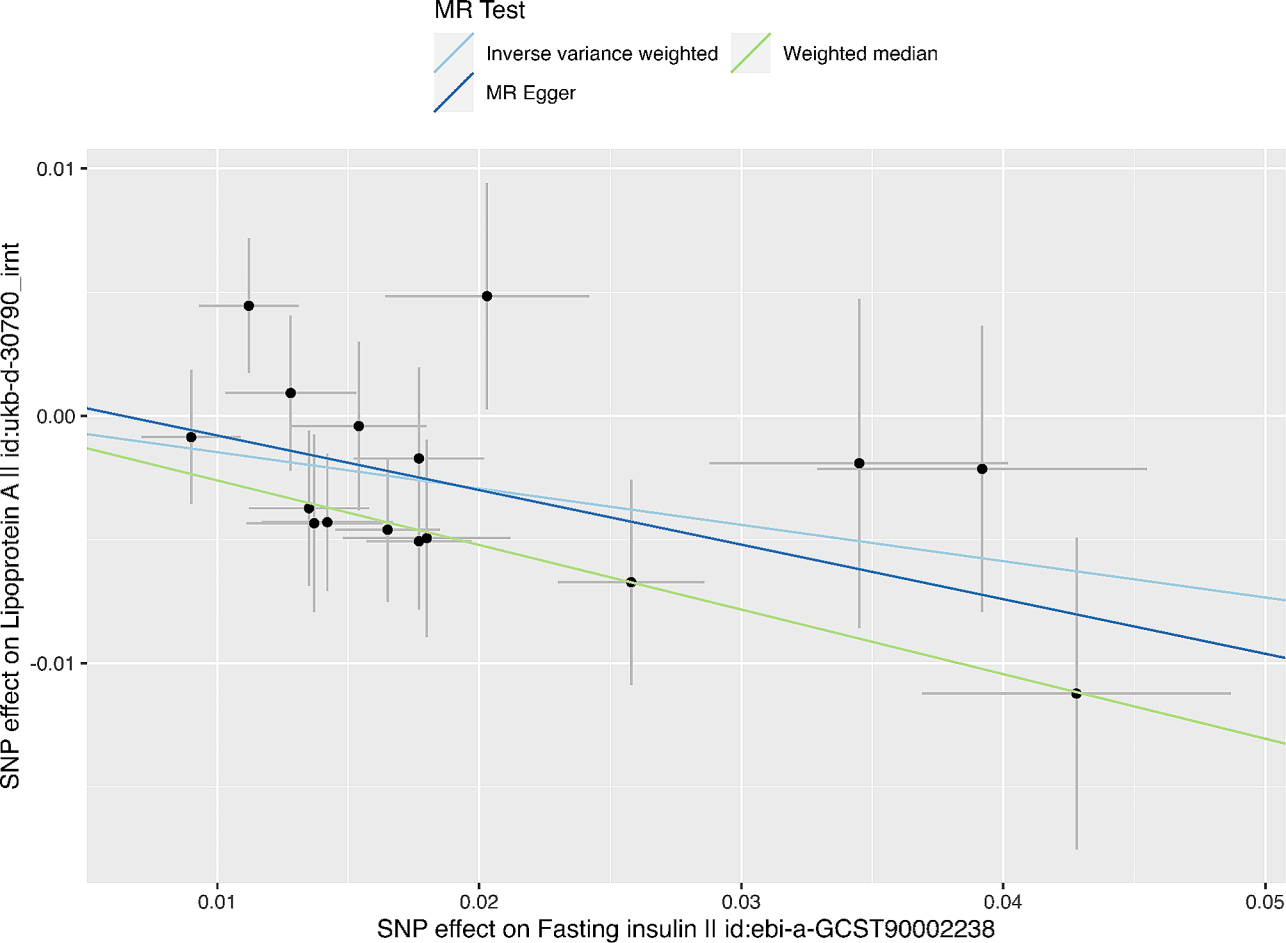
Genetic associations between fasting insulin and Lp(a). Each genetic variant included in the analysis is represented as a point + 95% CI. Location on the horizontal axis represents the correlation of the variant with exposure (plasma fasting insulin, inverse variance normal-transformed values); location on the vertical axis represents the correlation of the variant with the outcome (Lp(a), inverse-rank normalized). Lines represent estimates of different MR methods

**Fig. 2:**
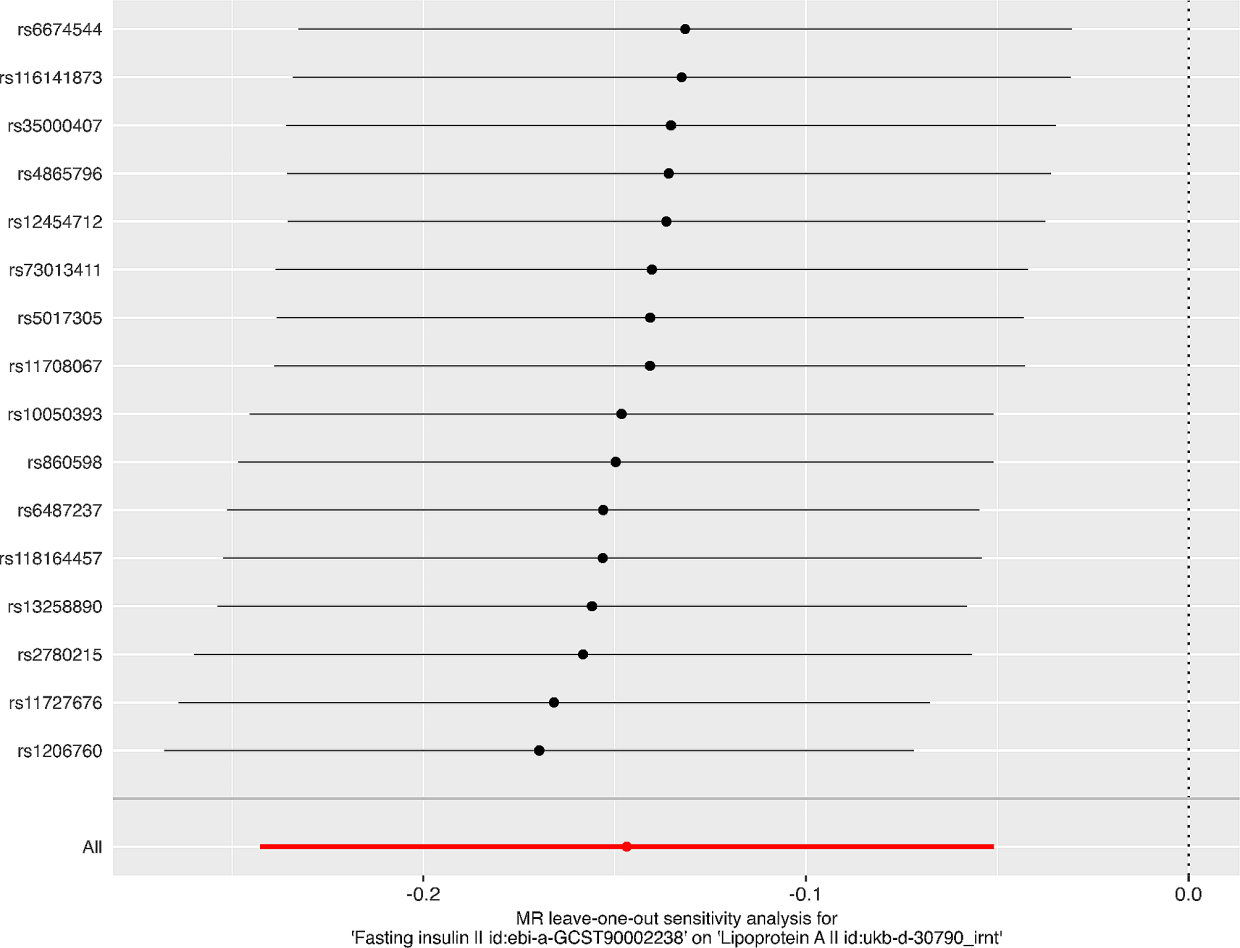
Results of leave-one-out analysis.  Horizontal axis—inverse-variance weighted-mean estimate with 95% confidence intervals of genetic associations between insulin and Lp(a). Each dot and confidence interval represents an inverse-variance weighted mean obtained when a variant listed on the left side is removed from the analysis.

As shown in Table 1; Fig. 1, the primary conservative analysis revealed higher genetically predicted fasting insulin levels to be associated with decreased Lp(a) levels (β = − 0.15, SE = 0.05, *P* = 0.003). Moreover, these findings were supported by the results of the liberal analysis (Supplementary Tables 8 and Supplementary Fig. 1, Additional file 3), in which genetically determined fasting insulin levels also influenced Lp(a) levels (β_IVM_ = −0.13, SE_IVM_ = 0.04, P_IVM_ = 0.0002; β_WME_ = −0.14, SE_WME_ = 0.05, P_WME_ = 0.005). In the sensitivity analysis, the results of the WME (β = −0.26, SE = 0.07; *P* = 0.0002) method supported this association. The MR‒Egger results were not significant (β = −0.22, SE = 0.13, *P* = 0.11) but yielded a consistent estimate. This is possibly due to the reduced power of MR‒Egger compared to IVM due to the additional intercept term. Based on Cochran’s Q test (P_IVM_ = 0.53, P_MR-Egger_ = 0.49) and MR-PRESSO analysis (*P* = 0.60), heterogeneity or outliers were not detected between the exposure and outcome genetic variants. Additionally, the MR‒Egger intercept test did not show evidence of horizontal pleiotropy (P_intercept_ = 0.54). The leave-one-out analysis indicated no significant change in the MR results after removing one SNP at a time (Fig. 2).

**Table 1:** Effect estimates of associations between fasting insulin (exposure) and Lp(a) (outcome) in conservative analysis

Outcome	Method	nSNP	β	SE	*P*	Cochran Q test *P*	MR‒Egger intercept (*P*)	Steiger test *P*	MR-PRESSO (Global *P* test)
Lp(a)	IVW	16	− 0.15	0.05	0.003	0.53	0.001 (0.54)	5.67e−78	0.60
	MR‒Egger	16	− 0.22	0.13	0.11	0.49			
	WME	16	− 0.26	0.07	0.0002				

Lp(a), lipoprotein(a); IVs, instrumental variables; nSNP, number of SNP; β, MR estimate; SE, standard error; *P*, *p*-value; IVW, inverse-variance weighted method; WME, the weighted median method; MR-PRESSO, MR pleiotropy residual sum and outlier

**Fig. 3:**
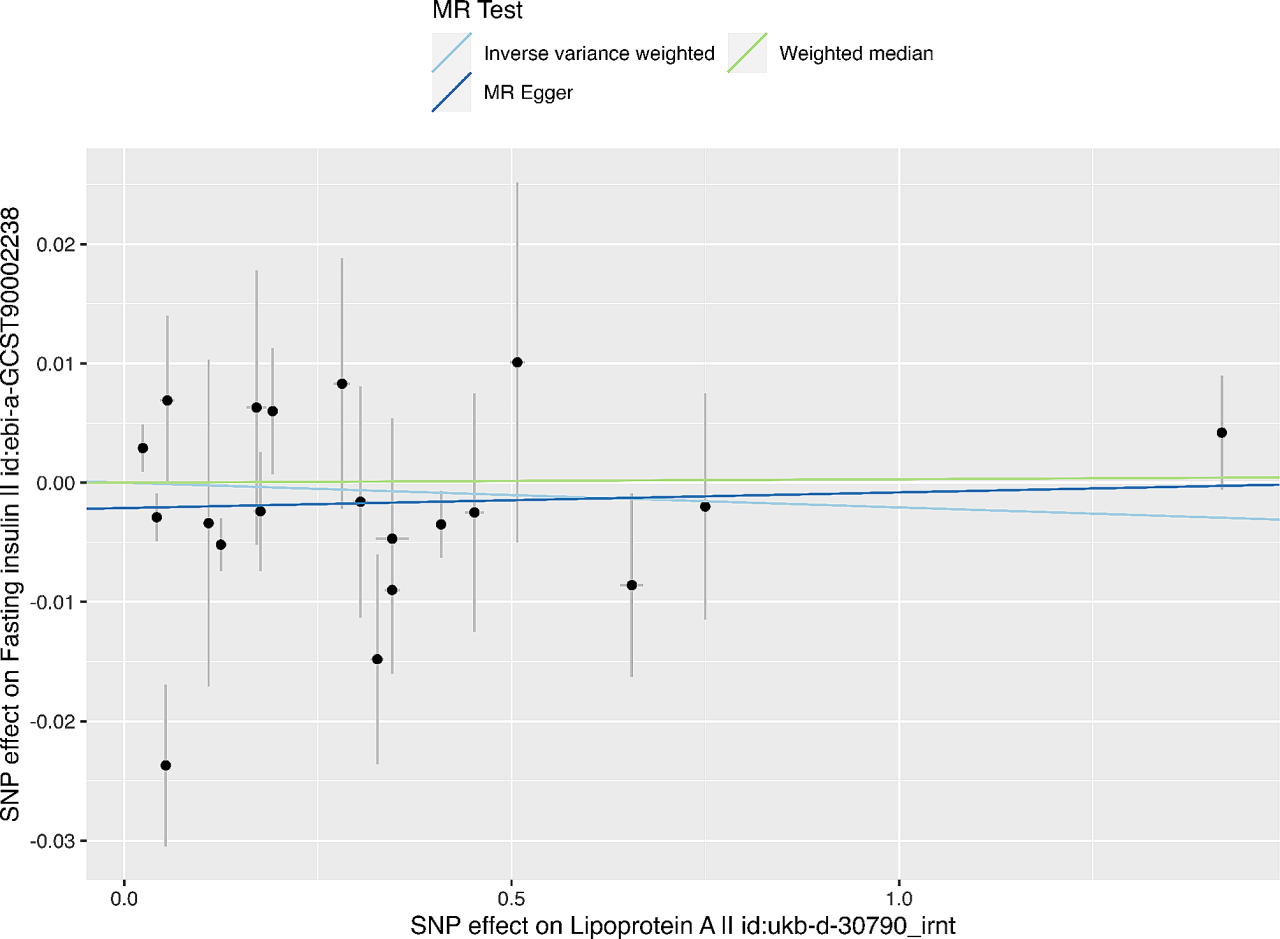
Genetic associations between Lp(a) levels and fasting insulin.Each genetic variant included in the analysis is represented as a point + 95% CI. Localization on the horizontal axis represents the correlation of the variant with exposure (Lp(a), inverse-rank normalized). Localization on the vertical axis represents the correlation of the variant with the outcome (plasma fasting insulin, inverse variance normal transformed values). Lines represent estimates of different MR methods.

Furthermore, no evidence of reverse causality was found between the exposure and outcome, as determined by the Steiger test (*P* = 5.67e−78, Table 1) and by conducting bidirectional MR (Table 2; Fig. 3).

**Table 2:** Effect estimates of the associations between genetic IVs for Lp(a) (exposure) and fasting insulin (outcome)

Outcome	Method	nSNP	β	SE	*P*	Cochran Q test *P*	MR-egger intercept (*P*)	MR-PRESSO (Global *P* test)
Insulin	IVW	20	−0.002	0.004	0.56	0.02		0.13
	MR-egger	20	0.001	0.004	0.76	0.04	−0.002 (0.17)	
	WME	20	0.0004	0.003	0.93			

IVs, instrumental variables; nSNP, number of SNP; β, MR estimate; SE, standard error; *P*, *p*-value; IVW, inverse-variance weighted method; WME, the weighted median method; MR-PRESSO, MR pleiotropy residual sum and outlier

**Fig. 4:**
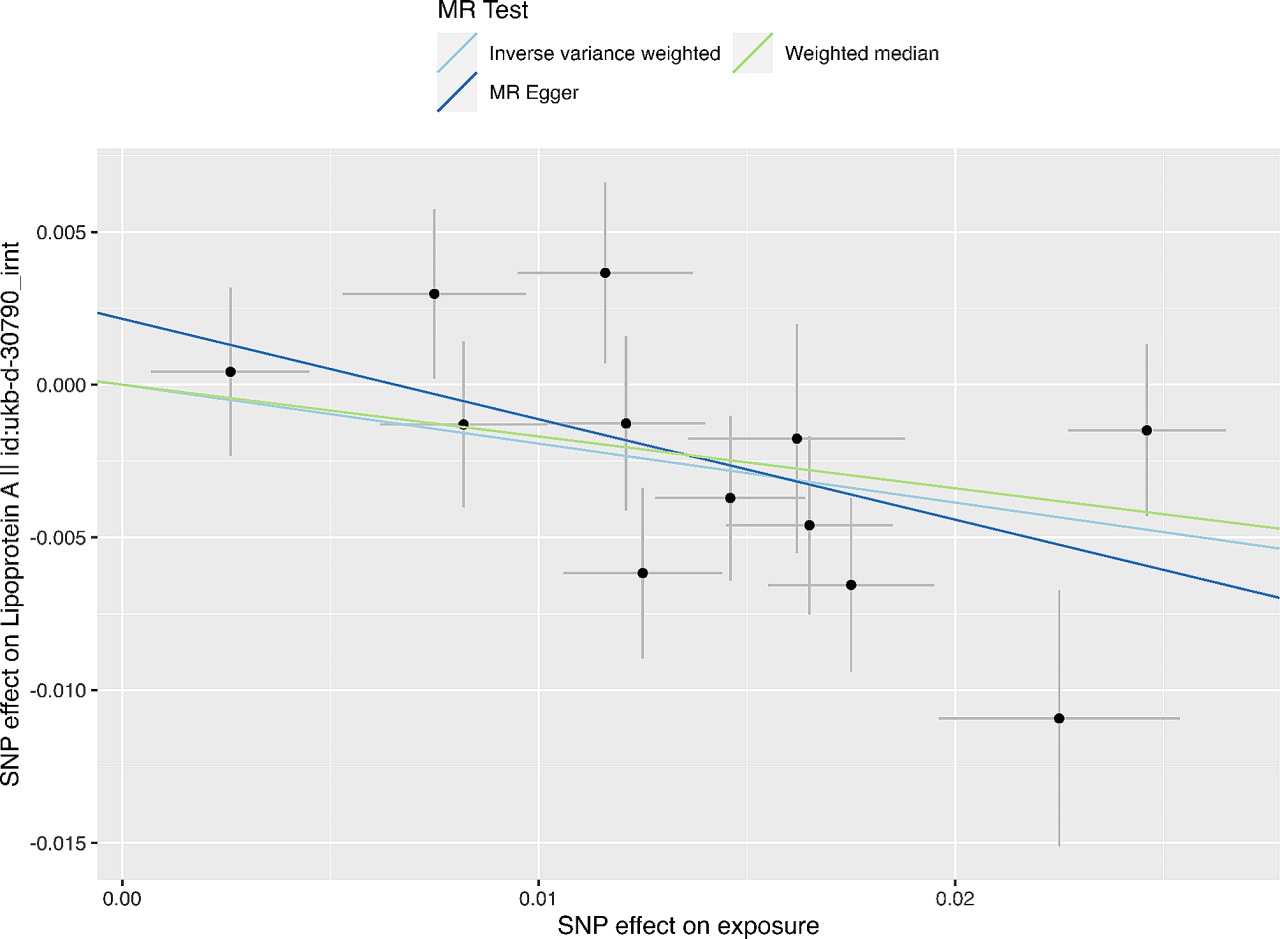
Genetic associations between fasting insulin levels and Lp(a) in additional sensitivity analysis. Each genetic variant included in the analysis is represented as a point + 95% CI. Localization on the horizontal axis represents the correlation of the variant with exposure (plasma fasting insulin, inverse variance normal transformed values). Localization on the vertical axis represents the correlation of the variant with the outcome (Lp(a), inverse-rank normalized). Lines represent estimates of different MR methods.

Additional analysis, which included IVs from Buchmann and colleagues’ paper [39], supported an association between genetically elevated fasting insulin and decreased Lp(a) by IVM (β = −0.19, SE = 0.07, *P* = 0.003) and WME (β = − 0.17, SE = 0.08, *P* = 0.04), but the results were not statistically significant by MR‒Egger (β = − 0.33, SE = 0.16, *P* = 0.07) method (Table 3; Fig. 4).

**Table 3:** Effect estimates of the associations between genetic IVs for insulin (exposure) and Lp(a) (outcome) in the additional sensitivity analysis

Outcome	Method	nSNP	β	SE	*P*	Cochran Q test *P*	MR-Egger Intercept (*P*)	MR-PRESSO (Global *P* Test)
Lp(a)	IVW	12	− 0.19	0.07	0.003	0.23		0.26
	MR-Egger	12	− 0.33	0.16	0.07	0.22	0.002 (0.38)	
	WME	12	− 0.17	0.08	0.04			

Lp(a), lipoprotein(a); IVs, instrumental variables; nSNP, number of SNP; β, MR estimate; SE, standard error; *P*, *p*-value; IVW, inverse-variance weighted method; WME, the weighted median method; MR-PRESSO, MR pleiotropy residual sum and outlier

We have also analysed the impact of fasting insulin on ApoB and LDL-C. Among 38 IV SNPs identified for fasting insulin, MR-PRESSO analysis has identified some outlier SNPs: 8 for LDL-C and 10 for ApoB. Full lists of SNPs excluded from the analysis for each parameter are presented in Supplementary Tables 9–10, Additional file 3. In the analysis of secondary Lp(a) components we have found that ApoB levels increased with fasting insulin levels (IVW: β = 0.24, SE = 0.004, *P* = 5.78e−08). The results of the WME (β = 0.27, SE = 0.05; *P* = 4.17e−07) and MR-Egger (β = 0.30, SE = 0.13; *P* = 2.53e−02) method supported this association (Supplementary Tables 11 and Supplementary Figs. 2–3, Additional file 3). LDL-C was found to increase due to an increase in genetic fasting insulin concentrations but only when the estimate was computed with IVW method (β = 0.09, SE = 0.04, *P* = 0.03) (Supplementary Tables 12 and Supplementary Figs. 4–5, Additional file 3). Notably, all analyses demonstrated significant heterogeneity in the IVs (Cochran Q test *P* < 0.05).

## Discussion

Our analysis revealed that an increase in the fasting insulin concentration causes a decrease in the Lp(a) concentration. It is known that hyperinsulinaemia is usually associated with the development of T2D. First, the emerging insulin resistance of peripheral tissues leads to excessive insulin synthesis, and both factors lead to prediabetes and then to T2D. In patients with T2D, insulin levels decrease as pancreatic beta cells become depleted [43]. Many studies have shown an inverse association between lower Lp(a) and insulin resistance [44, 45].

In vitro studies on the effect of insulin on Lp(a) synthesis have shown that high insulin concentrations reduce apo(a) synthesis. Such a relationship was indicated in a study conducted by Neele et al. based on cell culture of monkey hepatocytes. The study was motivated by the fact that elevated Lp(a) levels decreased after insulin therapy in patients with type 1 diabetes (T1D). The experiment showed that high insulin concentrations inhibit apo(a) synthesis at the posttranscriptional level, with the peak of inhibition occurring after 72 h of incubation of cells with insulin [23]. In another study published by Suzuki H et al. utilizing the HepG2 cell line, the researchers detected reduced apo(a) promoter activity due to the influence of insulin [22]. Importantly, MR tests chronic rather than short term exposures and this differentiates it from conventional in vitro studies. Unfortunately, there is a paucity of in vitro studies exploring the relationship between insulin and Lp(a) and further research in this area is necessary.

We would like to emphasize that, to the best of our knowledge, our MR study is the second analysis of this type published to date aiming to verify whether there are actual indications of regulation of Lp(a) concentration by insulin. Previous MR analyses typically concentrated on the causal impact of Lp(a) on T2D and did not produce fully consistent results [16–18].

The first previously published MR study was based on a meta-analysis of cross-sectional data for patients from three independent cohorts, i.e., Berlin Ageing Study II (BASE-II; N = 2012), LIFE-Adult (N = 3281) and LIFE-Heart (N = 2816), and used 2 or 12 IVs. That previous study comprised a one-sample MR analysis in which the genetic IVs risk factor associations and genetic IVs outcome associations were derived from the same sample, and individual-level data were used to derive MR estimates. In contrast to the results of our investigation, that study yielded no significant evidence of insulin-dependent regulation of Lp(a) concentration, although some analyses showed an insignificant tendency for insulin to reduce Lp(a) levels [39]. In our analysis, we performed two-sample MR on aggregated data, i.e., MR analyses in which the genetic IVs-risk factor associations and genetic IVs-outcome associations were (ideally) obtained from different (nonoverlapping) samples. Two-sample MR reduces the severity of “weak instrument bias”, “sample overlap”, and the appearance of “Winner’s curse” compared to one-sample MR, and the bias direction tends to approach null. The reliability of our results is primarily due to the type of analysis employed, the significantly larger group of subjects used, the larger number of IVs involved, and the more restrictive cut-off point for genome-wide significance applied (*p*-value < 5.00e−08). In addition, to validate our findings on a different IV set, we utilized variants used as insulin IVs in Buchmann and colleagues’ paper and performed MR, and we also obtained a result that partially supported our findings.

Interestingly, studies on acute exposure to exogenous insulin differ in their findings when compared to our MR analysis. An experimental study published by Sidhu et al. verified the relationship between serum Lp(a) concentration and insulin metabolism in healthy men without obesity undergoing an intravenous glucose tolerance test. Their study revealed a significant inverse relationship between the insulin level and Lp(a) concentration in the first, but not second phase of the plasma insulin response [46]. Another study conducted by Sutherland et al. evaluated the effect of changes in Lp(a) concentrations in T2D patients and healthy controls receiving intravenous insulin infusion. Plasma Lp(a) levels showed a small but significant increase in mean concentrations in T2D patients, but mean Lp(a) concentrations did not change significantly in healthy subjects [47]. In addition, two studies conducted by Riemens et al. are worth mentioning. In the first study, no Lp(a) concentration changes were observed when assessing the effects of 3-h hyperinsulinaemia induced by hyperglycaemia in healthy subjects [48]. In the second study, Riemes et al. verified the impact of 24-h intravenous insulin infusion on the Lp(a) concentration in plasma, and no significant changes in Lp(a) levels [49]. The results described above contrast with our observations, but it should be noted that MR analyses take into account long-term minor differences in exposure and not short-term, high-intensity interventions, as in the case of the results described by the researchers. The half-life of Lp(a) in human plasma is approximately 3 days [50]. Therefore, even several hours of insulin infusion in those studies may not have been long enough to detect significant differences in Lp(a) synthesis. This seems particularly likely considering the previously discussed results published by Neele et al., in which monkey hepatocytes showed a peak inhibition of Lp(a) synthesis under the influence of insulin only after 72 h [23]. It should be noted that, in comparison with the results of other research, our results are based on the MR, which is a method that enables estimating causal relationships in the observational study using genetic variants as instrumental variables. Our results are based on a large sample and the exclusion of diabetes as a confounding variable because these individuals are a heterogeneous group and may show different degrees of diabetes development.

As mentioned, MR analysis examines the effects of long-term changes in exposure over the lifespan rather than short-term effects. Studies determining the effects of chronic insulin therapy on Lp(a) levels yielded results more consistent with those of our MR analysis. In the first such study, Wolffenbuttel et al. examined the effect of improving blood glucose control with insulin therapy on Lp(a) and other lipoproteins in patients with T2D with poor disease control. It was concluded that insulin therapy does not alter Lp(a) levels in T2D patients after 6 months of insulin treatment [51]. Another study by Kuusi et al. aimed to verify the effect of insulin treatment on Lp(a) levels in T2D patients. Lp(a) concentration increased in patients with Lp(a) concentrations less than 300 mg/L but not in patients with Lp(a) concentrations greater than 300 mg/L. Interestingly, metabolic control was not related to the described effect [52]. Another study examined the effects of improved glycaemic control following 3 months of insulin therapy on Lp(a) concentration in patients with poorly controlled T2D. Lp(a) levels did not change, regardless of glycaemic control [53]. Although not a study of insulin administration but also an interventional study, a trial on 140 obese children (mean age 12.5 ± 1.6 years), demonstrated that a 3-week diet has significantly reduced both serum insulin and serum Lp(a) levels [54]. Once again, we would like to emphasize that in our analyses, the exposure dataset excluded patients with diabetes due to potential confounding due to disease treatment and progression. Our model reflects the dependence of Lp(a) on insulin in a physiological state and not during the progression and treatment of diabetes, when beta cells may eventually fail, insulin be of exogenous origin and insulin-sensitivity of tissues be modified. Additionally, in the case of individuals using insulin therapy, there may also be differences between insulin concentrations compared to the profile resulting from endogenous insulin secretion. These situations may impact the change in Lp(a) secretion. Therefore, in our opinion, caution should be taken when comparing the results of our analysis, which is based on the effect of insulin on Lp(a) concentration in non-diabetic individuals, with the results based on the assessment of insulin therapy in diabetes. 

The available results of cross-sectional studies aimed at establishing the relationship between insulin and Lp(a) concentrations also show divergent conclusions, but they are more in line with the results of our analysis. In a study conducted by Zamora-González et al. in the Mexican population, the fasting insulin concentration was found to be directly and independently inversely related to the Lp(a) level in patients of both sexes [55]. Another important study also concerning the Mexican population was conducted by Posadas-Romero et al., including included lean and obese subjects with normoglycaemia and normal blood pressure. Lp(a) concentrations were similar in women with obesity and high and normal concentrations of insulin (19.9 mg/dl vs. 18.6 mg/dl), but men with obesity and hyperinsulinaemia had significantly lower Lp(a) levels than did men with obesity and normo-insulinaemia (7.9 mg/dl vs. 29.4 mg/dl). These observations were not found in the female group [56]. On the other hand, in Jichi Medical Cohort Study, which included 1121 men and 1480 women, Inoue et al. showed a weak, statistically insignificant inverse correlation between insulin and Lp(a) in both men and women [57]. Another study by Habib et al. assessed the relationship between basal insulin concentration and Lp(a) concentration in a group of patients with and without T2D. The analysed groups were additionally divided into two subgroups based on normal (< 10 µU/mL) or elevated fasting insulin concentrations (≥ 10 µU/mL). The Lp(a) concentration was significantly lower in individuals with T2D with increased fasting insulin concentrations than in individuals with normal fasting insulin concentrations. Regression analysis confirmed that the Lp(a) concentration correlated inversely with the insulin concentration in individuals with T2D (*P* < 0.05) [58]. Another interesting study on Lp(a) and insulin concentrations in different types of diabetes was conducted by Heller et al. The study group consisted of patients with T1D and patients with T2D treated with diet, diet in combination with oral hypoglycaemic drugs, or requiring insulin therapy. The results of the analyses indicated that the concentration of Lp(a) in serum was not significantly related to the duration of diabetes in the individual or to the degree of glycaemic control. T2D patients receiving insulin therapy were observed to have increased serum Lp(a) concentrations [59]. A study conducted by Vaverkova et al. has found that in dyslipidemic patients there is a negative correlation between serum Lp(a) and insulin [60]. Finally, in a study of 7633 Chinese men, patients with insulin resistance were found to exhibit both higher insulin levels and lower Lp(a) levels [44]. On the other hand, the positive correlation between fasting insulin and Lp(a) was noted in a sample of 131 normal-weight Mexican children with an average age of 7.7 ± 0.8 years. It was reported that children with hyperinsulinemia had significantly increased Lp(a) levels [61]. It may be possible that factors affecting Lp(a) and insulin relationship are different in pre-pubertal children. In summary, several observational studies may suggest that endogenous fasting insulin is indeed negatively associated with Lp(a).

Overall, the results of studies published thus far indicate that endogenous insulin has a slight tendency to reduce Lp(a) concentrations. In contrast, acute administration of exogenous insulin may have the opposite effect or have no significant impact on Lp(a) concentration. In this respect, the previously discussed in vitro study by Neele et al. may be difficult to classify as either acute or chronic exposure, but in that case, the exposure of liver cells to insulin was also more long-lasting and constant than in the case of bolus insulin therapy or an infusion of this hormone lasting a mere few hours and the study explored apo(a) expression rather than simple Lp(a) levels. As already mentioned, our MR study examined long-term changes in exposure to endogenous insulin. Hence, the results of the observational studies seem consistent with MR results, suggesting a negative albeit weak effect of insulin concentration on plasma Lp(a) concentrations.

Lp(a) levels appear to be related to sex and sex hormones. The literature is not fully consistent in terms of the correlation between gender and Lp(a) levels. An increased serum Lp(a) levels were found in diabetic females compared to males and non-diabetic female, regardless of menopausal status [62], in white women but not in black women [63], while in a different study no significant differences between males and females in regards to Lp(a) category were detected [64]. Hormone replacement therapy was found to lower Lp(a) concentration in post-menopausal women [65, 66]. Interestingly, a decrease in Lp(a) concentrations was also found in healthy males after testosterone administration [67, 68] but not in post-menopausal women [69]. On the other hand, no correlation between Lp(a) and sex hormone concentrations was found in a study of Finnish and American men [70], while in the study of Italian males, only a negative correlation between Lp(a) and DHEA-S was found [71]. In one study, Lp(a) was reported to be positively correlated with estradiol concentrations but only in women with self-reported heart disease [72].

In our study, we have also analysed the impact of fasting insulin levels on components of Lp(a) different than apo(a), namely ApoB and LDL-cholesterol, which was chosen because Lp(a) lipid core mostly resembles that of LDL particles. Lipoprotein(a) assembly mechanism does not appear to be clearly elucidated, but some have suggested ApoB to be a limiting factor based on studies in subjects with abetalipoproteinemia [73, 74]. However, we have found ApoB levels to increase with insulin levels which is the exact opposite of the expected effect if fasting insulin decreased Lp(a) through reducing ApoB availability. Interestingly, other MR analyses have found ApoB to be either unaffected by fasting insulin levels or to be increased by BMI-adjusted insulin levels, like ones used in our study [24, 25]. This finding contrasts with the results of in vitro and in vivo studies [75–78][ It may be possible that in our study the increase in ApoB due to elevated fasting insulin reflects metabolic dysfunction and insulin resistance rather than simple direct action of insulin. The results regarding the influence of fasting insulin on LDL-C were not fully consistent. Some have found that fasting insulin only affects some LDL fractions in terms of their composition [24], which may explain this phenomenon.

Our analysis concluded that increased serum insulin concentration may contribute to a decrease in Lp(a) concentration. Nevertheless, it is important to acknowledge some limitations of this study. This study relied on publicly accessible summary-level data, and the available data precluded us from performing additional subgroup analyses to explore the influence of nongenetic factors affecting Lp(a) levels, such as sex, hormones, or diet. Moreover, the exposure data excluded patients with diabetes, which raises the possibility of selection bias and collider bias [79], although insulin levels in patients with diabetes can also be affected by treatment and disease progression [80]. Additionally, the study was limited to individuals of European descent and did not distinguish between males and females, raising questions about the generalizability of our findings to populations of different ethnicities and genders. Finally, insulin and glycemia-related traits are highly complex and interconnected. Further analyses are required to determine if there exist indirect effects of insulin on Lp(a) levels due to other mechanisms related to insulin resistance rather than direct action of insulin.

There are two clinical implications of this study. First, it is clear that the relationship between Lp(a) and diabetes is not trivial and that Lp(a) is likely not a risk factor for the development of diabetes independent of preexisting hyperinsulinaemia and insulin resistance. Second, the observational relationship between low Lp(a) and diabetes risk may not translate to adverse effects of therapies that reduce Lp(a) levels due to causal relationships. Although therapies aimed at reducing insulin resistance, insulinaemia and glycaemia may increase Lp(a), it is almost certain that their cardiometabolic benefits outweigh the increased cardiovascular risk caused by an increase in Lp(a) as demonstrated by the fact that good glycemic control improves patient survival [81].

## Conclusion

The results of our MR study support evidence that elevated insulin concentrations may be causally related to decreased serum Lp(a) levels. Our findings suggest that hyperinsulinaemia, which typically accompanies T2D [82], can partially explain the inverse relationship between low Lp(a) concentrations and an increased risk of T2D. Further studies are needed to validate and establish the exact mechanism behind this relationship.

### Supplementary material

Additional file 1.

Additional file 2.

Additional file 3.

## Data Availability

The statistical code used in this work is available upon reasonable request to the corresponding author. Access to the GWAS summary statistics was obtained through the OpenGWAS project developed at the MRC Integrative Epidemiology Unit (IEU) [26]. The GWAS ID corresponding to insulin was “ebi-a-GCST90002238”. The GWAS ID corresponding to Lp(a) was “ukb-d-30790_irnt”. No original raw data were generated in this study.

## Abbreviations

apo(a): Apolipoprotein(a)
apoB: Apolipoprotein B100
CVD: Cardiovascular diseases
EAF: Effect allele frequency
GWASs: Genome-wide association studies
IVs: Instrument variables
IVW: Inverse-variance weighted
KIV-2: Kringle-IV 2
LD: Linkage disequilibrium
Lp(a): Lipoprotein(a)
MR: Mendelian randomization
T1D: Type 1 diabetes
T2D: Type 2 diabetes
WME: Weighted median
MR-PRESSO: MR pleiotropy residual sum and outlier
SNP: Single-nucleotide polymorphism
STROBE: Strengthening the reporting of observational studies in epidemiology
