# Supplementary material for: Causal associations between insulin and Lp(a) levels in Caucasian population: a Mendelian randomization study
Source: Cardiovasc Diabetol. 2024 Aug 29;23:316. doi: 10.1186/s12933-024-02389-7 (PMC11360791; doi:10.1186/s12933-024-02389-7)
Supplement: Supplementary file 3 — Additional file 3. [file 12933_2024_2389_MOESM3_ESM.docx]

Supplementary Table 5. Characteristics of IVs for insulin in the conservative analysis

| **SNP** | **Chr** | **Position** | **EA** | **OA** | **EAF** | **Beta** | **SE** | **P** | **R2** | **F statistic** |
| --- | --- | --- | --- | --- | --- | --- | --- | --- | --- | --- |
| **rs6674544** | 1 | 219628973 | A | G | 0.02 | 0.002 | 0.57 | 6.97e-21 | 0.0005 | 78 |
| **rs5017305** | 2 | 630902 | T | A | -0.01 | 0.003 | 0.76 | 1.07e-08 | 0.0002 | 28 |
| **rs35000407** | 3 | 12351521 | G | T | -0.03 | 0.003 | 0.12 | 1.50e-21 | 0.0006 | 85 |
| **rs11708067** | 3 | 123065778 | G | A | 0.01 | 0.002 | 0.18 | 1.30e-09 | 0.0002 | 34 |
| **rs11727676** | 4 | 145659064 | C | T | 0.02 | 0.004 | 0.08 | 2.90e-08 | 0.0002 | 27 |
| **rs4865796** | 5 | 53272664 | A | G | 0.02 | 0.002 | 0.71 | 7.33e-17 | 0.0005 | 68 |
| **rs10050393** | 5 | 157918946 | C | T | -0.01 | 0.002 | 0.46 | 4.84e-08 | 0.0001 | 22 |
| **rs73013411** | 6 | 164126233 | A | C | -0.02 | 0.003 | 0.12 | 2.08e-08 | 0.0002 | 32 |
| **rs2780215** | 6 | 34236973 | G | A | -0.04 | 0.01 | 0.04 | 1.06e-09 | 0.0003 | 39 |
| **rs116141873** | 6 | 34222201 | T | G | 0.04 | 0.01 | 0.03 | 1.42e-11 | 0.0003 | 53 |
| **rs13258890** | 8 | 23615445 | C | T | -0.01 | 0.003 | 0.25 | 2.77e-08 | 0.0002 | 26 |
| **rs118164457** | 10 | 89680631 | C | T | 0.03 | 0.01 | 0.04 | 3.86e-10 | 0.0002 | 37 |
| **rs6487237** | 12 | 21699928 | A | C | 0.02 | 0.003 | 0.79 | 4.68e-09 | 0.0002 | 35 |
| **rs860598** | 12 | 102898446 | A | G | 0.02 | 0.003 | 0.82 | 6.88e-12 | 0.0003 | 50 |
| **rs12454712** | 18 | 60845884 | C | T | -0.01 | 0.003 | 0.40 | 1.78e-09 | 0.0002 | 32 |
| **rs1206760** | 20 | 45582472 | A | G | -0.01 | 0.002 | 0.52 | 8.82e-10 | 0.0002 | 35 |

#### Abbreviations: IVs - instrumental variables; SNP - single nucleotide polymorphism; Chr - chromosome; EA - effect alleles; OA - other alleles; EAF - effect allele frequency; SE - standard error; P - p-value; R2 - variance

Supplementary Table 6. Characteristics of added IVs for insulin in the liberal analysis

| **SNP** | **Chr** | **Position** | **EA** | **OA** | **EAF** | **Beta** | **SE** | **P** | **R2** | **F statistic** |
| --- | --- | --- | --- | --- | --- | --- | --- | --- | --- | --- |
| **rs13389219** | 2 | 165528876 | T | C | 0.40 | -0.02 | 0.002 | 5.84e-28 | 0.001 | 110 |
| **rs2943646** | 2 | 227099534 | G | A | 0.62 | 0.03 | 0.002 | 8.47e-39 | 0.001 | 173 |
| **rs1260326** | 2 | 27730940 | C | T | 0.59 | 0.004 | 0.002 | 8.42e-38 | 0.001 | 148 |
| **rs17036126** | 3 | 12287863 | T | C | 0.13 | 0.02 | 0.003 | 1.28e-10 | 0.0003 | 49 |
| **rs62271373** | 3 | 150066540 | A | T | 0.06 | 0.03 | 0.005 | 1.60e-08 | 0.0002 | 28 |
| **rs17331151** | 3 | 52844534 | T | C | 0.11 | -0.02 | 0.003 | 1.52e-08 | 0.0002 | 27 |
| **rs10865959** | 3 | 49891002 | C | G | 0.30 | 0.01 | 0.002 | 1.99e-08 | 0.0003 | 39 |
| **rs3775380** | 4 | 89739808 | G | A | 0.50 | 0.01 | 0.002 | 1.48e-11 | 0.0003 | 44 |
| **rs9884482** | 4 | 106081636 | C | T | 0.39 | 0.01 | 0.002 | 2.88e-11 | 0.0003 | 43 |
| **rs6855363** | 4 | 157670537 | C | T | 0.347 | -0.01 | 0.002 | 4.04e-08 | 0.0003 | 39 |
| **rs10050393** | 5 | 157918946 | C | T | 0.46 | -0.009 | 0.002 | 4.84e-08 | 0.0001 | 22 |
| **rs1474696** | 6 | 127449246 | G | A | 0.45 | 0.02 | 0.002 | 3.02e-16 | 0.0004 | 67 |
| **rs6905288** | 6 | 43758873 | A | G | 0.60 | 0.01 | 0.002 | 7.75e-09 | 0.0002 | 35 |
| **rs2108349** | 7 | 50786663 | A | G | 0.69 | -0.01 | 0.002 | 1.13e-08 | 0.0002 | 33 |
| **rs972283** | 7 | 130466854 | G | A | 0.54 | 0.01 | 0.002 | 1.09e-08 | 0.0002 | 30 |
| **rs7012814** | 8 | 9173358 | A | G | 0.47 | -0.02 | 0.002 | 8.34e-30 | 0.0009 | 133 |
| **rs75179845** | 9 | 136132954 | C | T | 0.08 | 0.02 | 0.004 | 6.05e-11 | 0.0003 | 38 |
| **rs7903146** | 10 | 114758349 | T | C | 0.31 | -0.01 | 0.002 | 1.24e-09 | 0.0002 | 30 |
| **rs2845885** | 11 | 63869062 | T | C | 0.93 | -0.02 | 0.004 | 1.18e-08 | 0.0002 | 27 |
| **rs1351394** | 12 | 66351826 | C | T | 0.53 | 0.01 | 0.002 | 2.71e-09 | 0.0003 | 38 |
| **rs7133378** | 12 | 124409502 | A | G | 0.34 | -0.01 | 0.002 | 6.00e-11 | 0.0003 | 40 |
| **rs731839** | 19 | 33899065 | A | G | 0.66 | -0.01 | 0.002 | 3.87e-11 | 0.0003 | 41 |

#### Abbreviations: IVs - instrumental variables; SNP - single nucleotide polymorphism; Chr - chromosome; EA - effect alleles; OA - other alleles; EAF - effect allele frequency; SE - standard error; P - p-value; R2 - variance

Supplementary Table 7. Characteristics of IVs for insulin in the additional sensitivity analysis

| **SNP** | **Chr** | **Position** | **EA** | **OA** | **EAF** | **Beta** | **SE** | **P** | **R2** | **F statistic** |
| --- | --- | --- | --- | --- | --- | --- | --- | --- | --- | --- |
| **rs2820436** | 1 | 219640680 | A | C | 0.32 | -0.02 | 0.002 | 4.80e-19 | 0.0001 | 77 |
| **rs1530559** | 2 | 135755629 | A | G | 0.46 | 0.008 | 0.002 | 9.47e-04 | 0.00003 | 12 |
| **rs2972143** | 2 | 227116365 | A | G | 0.37 | -0.03 | 0.002 | 2.00e-36 | 0.0003 | 168 |
| **rs9884482** | 4 | 106081636 | T | C | 0.61 | -0.01 | 0.002 | 2.88e-11 | 0.0001 | 43 |
| **rs4865796** | 5 | 53272664 | A | G | 0.71 | 0.02 | 0.002 | 7.33e-17 | 0.0001 | 68 |
| **rs2745353** | 6 | 127452935 | T | C | 0.48 | 0.02 | 0.002 | 4.63e-16 | 0.0001 | 66 |
| **rs1167800** | 7 | 75176196 | A | G | 0.55 | 0.008 | 0.002 | 5.59e-05 | 0.00003 | 17 |
| **rs983309** | 8 | 9177732 | T | G | 0.10 | 0.02 | 0.003 | 1.11e-17 | 0.0001 | 60 |
| **rs7903146** | 10 | 114758349 | T | C | 0.31 | -0.01 | 0.002 | 1.24e-09 | 0.0001 | 31 |
| **rsS35767** | 12 | 102875569 | A | G | 0.16 | -0.02 | 0.003 | 6.99e-10 | 0.0001 | 39 |
| **rs1421085** | 16 | 53800954 | T | C | 0.56 | 0.003 | 0.002 | 2.77e-01 | 0.000003 | 2 |
| **rs731839** | 19 | 33899065 | A | G | 0.66 | -0.012 | 0.002 | 3.87e-11 | 0.0001 | 41 |

#### Abbreviations: IVs - instrumental variables; SNP - single nucleotide polymorphism; Chr - chromosome; EA - effect alleles; OA - other alleles; EAF - effect allele frequency; SE - standard error; P - p-value; R2 - variance

#### Supplementary Table 8. Effect estimates of the associations between genetic IVs for insulin (exposure) and Lp(a) (outcome) in liberal analysis

| **Outcome** | **Method** | **nSNP** | **β** | **SE** | **P** | **Cochran Q** **test P** | **MR-egger**  **Intercept (P)** | **Steiger**  **test P** | **MR-PRESSO**  **(Global P Test)** |
| --- | --- | --- | --- | --- | --- | --- | --- | --- | --- |
| **Lp(a)** | IVW | 38 | -0.13 | 0.04 | 0.0002 | 0.08 |  | 2.60e-217 | 0.09 |
|  | MR-egger | 38 | -0.19 | 0.11 | 0.09 | 0.07 | 0.0009 (0.62) |  |  |
|  | WME | 38 | -0.14 | 0.05 | 0.005 |  |  |  |  |

##### Abbreviations: Lp(a) - lipoprotein(a); IVs - instrumental variables; nSNP - number of SNP; β - MR estimate; SE - standard error ; P - p-value; IVW - inverse-variance weighted method; WME - the weighted median method

#####
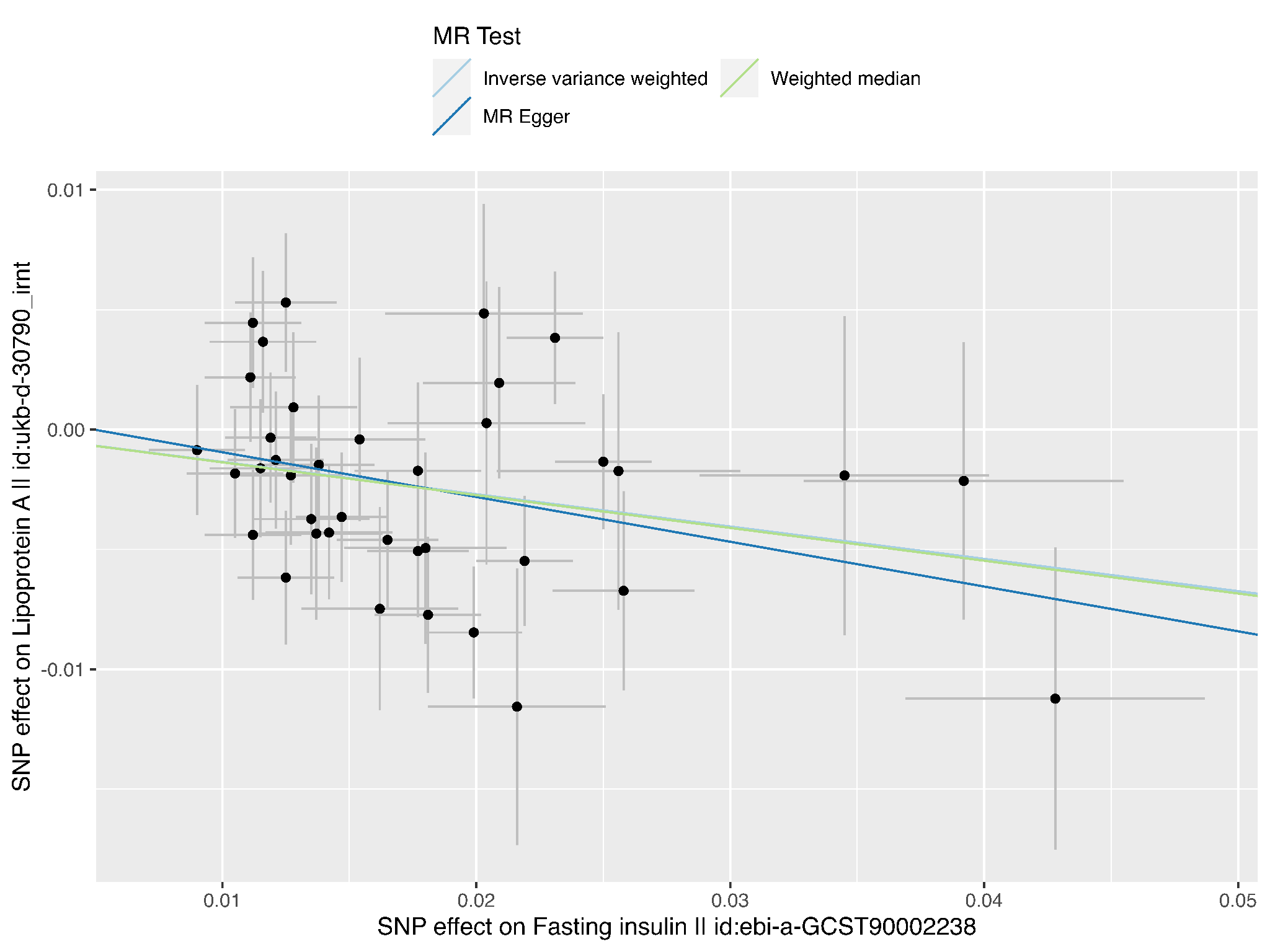


##### Supplementary Figure 1. Genetic associations between fasting insulin levels and Lp(a) in liberal analysis.

##### Supplementary Figure 1 Legend. Each genetic variant included in the analysis is represented as a point + 95% CI. Localization on the horizontal axis represents the correlation of the variant with exposure (plasma fasting insulin. inverse variance normal transformed values). Localization on the vertical axis represents the correlation of the variant with the outcome (Lp(a), inverse-rank normalized). Lines represent estimates of different MR methods.

Supplementary Table 9. Pleiotropic SNPs excluded from the liberal analysis (ApoB)

| **SNP** | **Chr** | **Position** | **Gene** | **EA** | **OA** | **EAF** | **Beta** | **SE** | **P** |
| --- | --- | --- | --- | --- | --- | --- | --- | --- | --- |
| **rs1260326** | 2 | 27730940 | GCKR | C | T | 0.59 | 0.02 | 0.002 | 8.42e-38 |
| **rs13389219** | 2 | 165528876 | COBLL1 | T | C | 0.41 | -0.02 | 0.002 | 5.84e-28 |
| **rs17331151** | 3 | 52844534 | LOC124906240 | T | C | 0.11 | -0.02 | 0.003 | 1.52e-08 |
| **rs1474696** | 6 | 127449246 | RSPO3 | G | A | 0.48 | 0.01 | 0.002 | 3.02e-16 |
| **rs6905288** | 6 | 43758873 | intergenic | A | G | 0.60 | 0.01 | 0.002 | 7.75e-09 |
| **rs972283** | 7 | 130466854 | COBLL1 | G | A | 0.54 | 0.01 | 0.002 | 1.09e-08 |
| **rs7012814** | 8 | 9173358 | DNAH10 | A | G | 0.47 | -0.02 | 0.002 | 8.34e-30 |
| **rs75179845** | 9 | 136132954 | TCF7L2 | C | T | 0.08 | 0.02 | 0.004 | 6.05e-11 |
| **rs7903146** | 10 | 114758349 | GCKR | T | C | 0.31 | -0.01 | 0.002 | 1.24e-09 |
| **rs7133378** | 12 | 124409502 | ABO | A | G | 0.34 | -0.01 | 0.002 | 6.00e-11 |

Abbreviations: SNP - single nucleotide polymorphism; Chr - chromosome; EA - effect alleles; OA - other alleles; EAF - effect allele frequency; SE - standard error; P - p-value

Supplementary Table 10. Pleiotropic SNPs excluded from the liberal analysis (LDL-C)

| **SNP** | **Chr** | **Position** | **Gene** | **EA** | **OA** | **EAF** | **Beta** | **SE** | **P** |
| --- | --- | --- | --- | --- | --- | --- | --- | --- | --- |
| **rs1260326** | 2 | 27730940 | GCKR | C | T | 0.59 | 0.02 | 0.002 | 8.42e-38 |
| **rs13389219** | 2 | 165528876 | COBLL1 | T | C | 0.41 | -0.02 | 0.002 | 5.84e-28 |
| **rs17331151** | 3 | 52844534 | LOC124906240 | T | C | 0.11 | -0.02 | 0.003 | 1.52e-08 |
| **rs1474696** | 6 | 127449246 | RSPO3 | G | A | 0.48 | 0.01 | 0.002 | 3.02e-16 |
| **rs7012814** | 8 | 9173358 | intergenic | A | G | 0.47 | -0.02 | 0.002 | 8.34e-30 |
| **rs75179845** | 9 | 136132954 | ABO | C | T | 0.08 | 0.02 | 0.004 | 6.05e-11 |
| **rs7903146** | 10 | 114758349 | TCF7L2 | T | C | 0.31 | -0.01 | 0.002 | 1.24e-09 |
| **rs7133378** | 12 | 124409502 | DNAH10 | A | G | 0.34 | -0.01 | 0.002 | 6.00e-11 |

Abbreviations: SNP - single nucleotide polymorphism; Chr - chromosome; EA - effect alleles; OA - other alleles; EAF - effect allele frequency; SE - standard error; P - p-value

Supplementary Table 11. Effect estimates of the associations between genetic IVs for insulin (exposure) and ApoB (outcome) in liberal analysis (outlier SNPs excluded)

| **Outcome** | **Method** | **nSNP** | **β** | **SE** | **P** | **Cochran Q**  **test P** | **MR-Egger**  **Intercept (P)** | **Steiger**  **test P** |
| --- | --- | --- | --- | --- | --- | --- | --- | --- |
| **ApoB** | IVW | 28* | 0.24 | 0.004 | 5.78e-08 | 0.01 |  | 8.41e-137 |
|  | MR-Egger | 28* | 0.30 | 0.13 | 2.53e-02 | 0.004 | -0.001 (0.62) |  |
|  | WME | 28* | 0.27 | 0.05 | 4.17e-07 |  |  |  |

Abbreviations: apoB - l apolipoprotein B; IVs - instrumental variables; nSNP - number of SNP; β - MR estimate; SE - standard error ;
P - p-value; IVW - inverse-variance weighted method; WME - the weighted median method
*Using the Mendelian Randomization Pleiotropy RESidual Sum and Outlier (MR-PRESSO) method, SNPs responsible for horizontal pleiotropy were detected in the liberal MR analysis. The outlier SNPs (rs1260326, rs13389219, rs1474696, rs17331151, rs6905288, rs7012814, rs7133378, rs75179845, rs7903146, rs972283) were removed from this analysis.


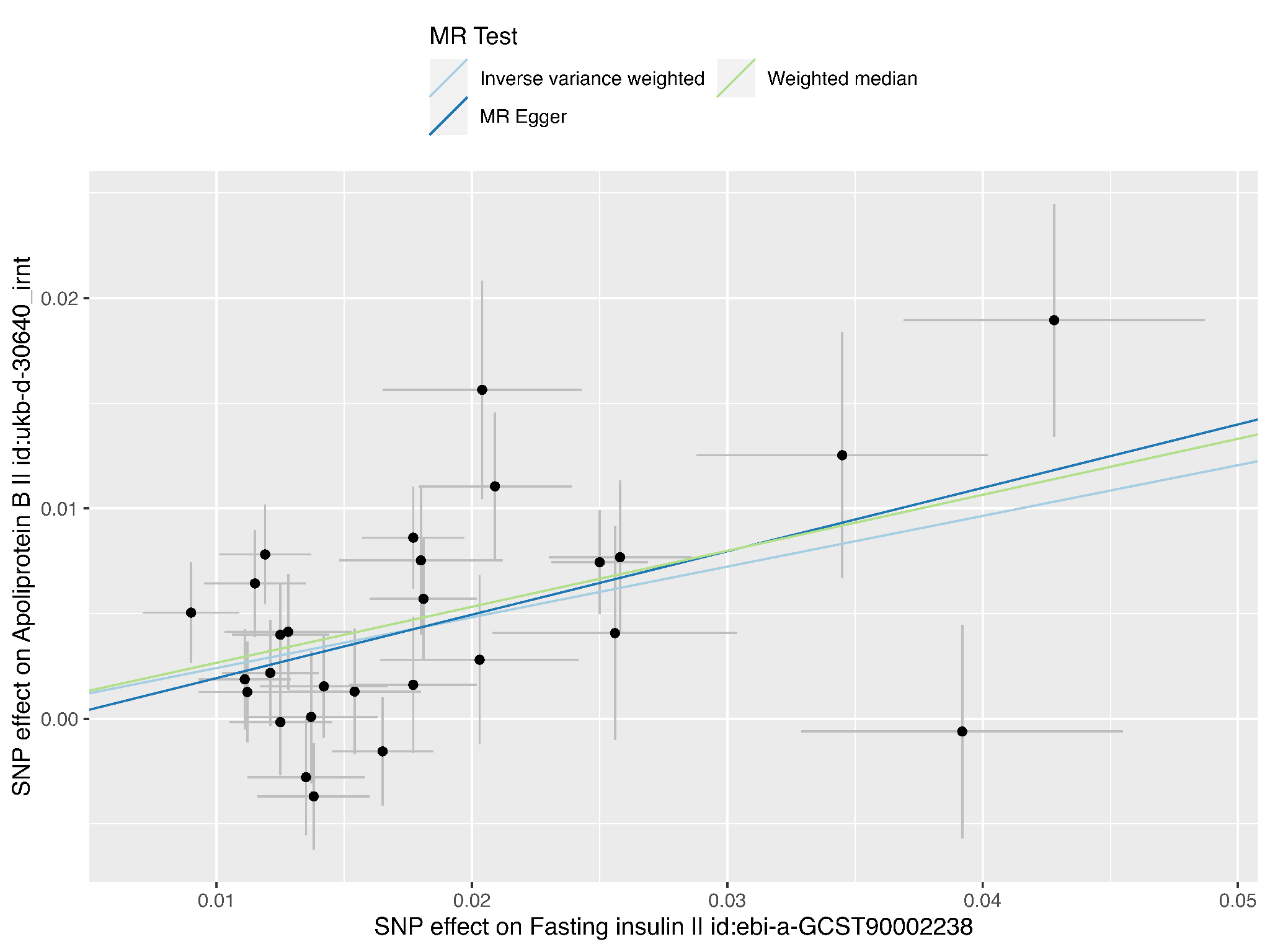


Supplementary Figure 2. Genetic associations between fasting insulin levels and ApoB in liberal analysis (outlier SNP excluded)

Supplementary Figure 2 Legend. Each genetic variant included in the analysis is represented as a point + 95% CI. Localization on the horizontal axis represents the correlation of the variant with exposure (plasma fasting insulin, inverse variance normal transformed values). Localization on the vertical axis represents the correlation of the variant with the outcome (ApoB, inverse-rank normalized). Lines represent estimates of different MR methods.


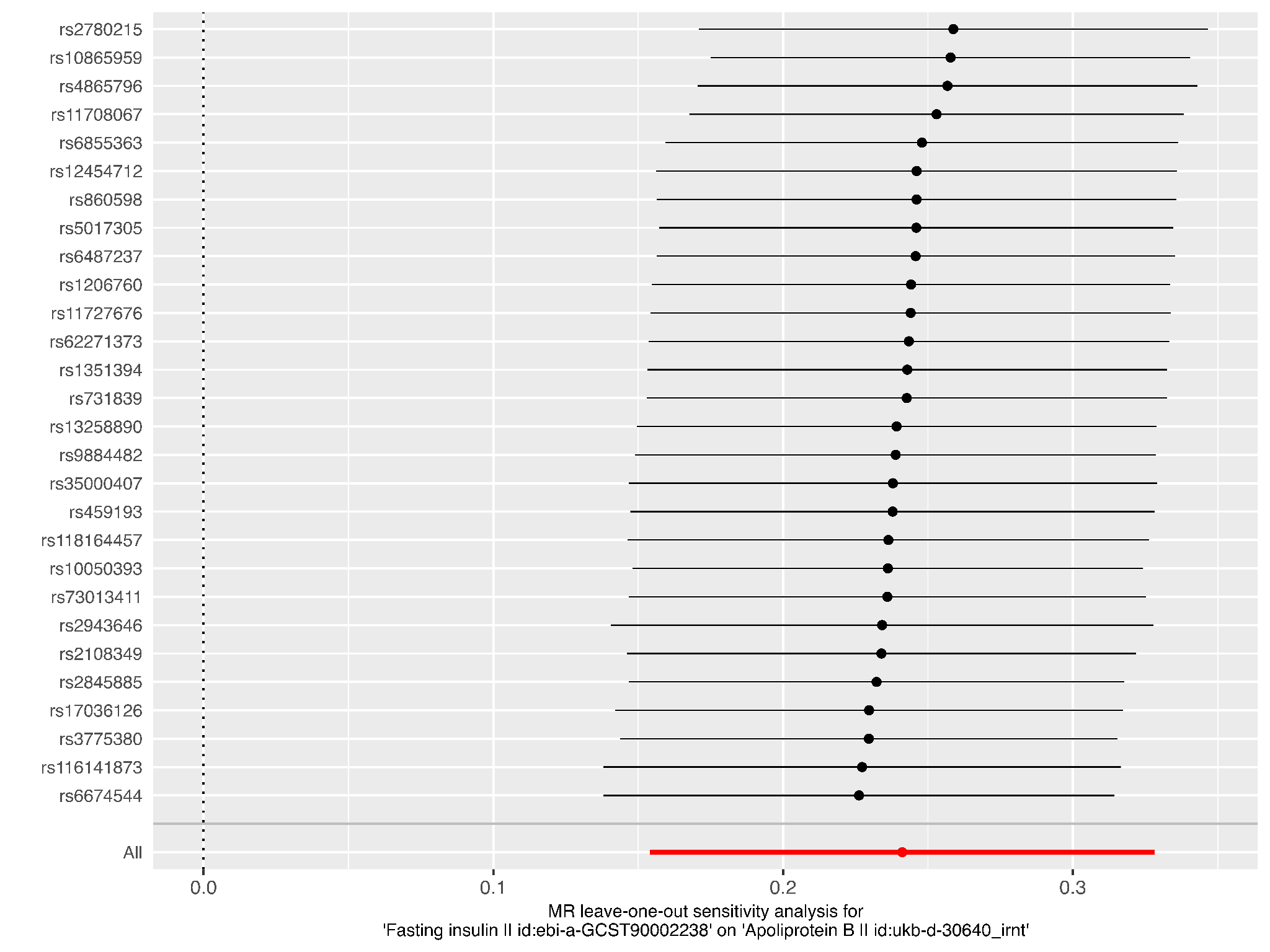


Supplementary Figure 3. Results of leave-one-out analysis
Supplementary Figure 3 Legend: Horizontal axis – inverse-variance weighted-mean estimate with 95% confidence intervals of genetic associations between insulin and ApoB. Each dot and confidence interval represents an inverse-variance weighted mean obtained when a variant listed on the left side is removed from the analysis.

Supplementary Table 12. Effect estimates of the associations between genetic IVs for insulin (exposure) and LDL-C (outcome) in liberal analysis (outlier SNPs excluded)

| **Outcome** | **Method** | **nSNP** | **β** | **SE** | **P** | **Cochran Q**  **test P** | **MR-Egger**  **Intercept (P)** | **Steiger**  **test P** |
| --- | --- | --- | --- | --- | --- | --- | --- | --- |
| **LDL-C** | IVW | 30* | 0.09 | 0.04 | 0.03 | 0.01 |  | 2.95e-162 |
|  | MR-Egger | 30* | -0.02 | 0.12 | 0.87 | 0.02 | 0.002 (0.32) |  |
|  | WME | 30* | 0.03 | 0.05 | 0.59 |  |  |  |

Abbreviations: LDL - low-density lipoprotein; IVs - instrumental variables; nSNP - number of SNP; β - MR estimate; SE - standard error ;
P - p-value; IVW - inverse-variance weighted method; WME - the weighted median method
*Using the Mendelian Randomization Pleiotropy RESidual Sum and Outlier (MR-PRESSO) method, SNPs responsible for horizontal pleiotropy were detected in the liberal MR analysis. The outlier SNPs (rs1260326, rs13389219, rs1474696, rs17331151, rs7012814, rs7133378, rs75179845, rs7903146) were removed from this analysis.


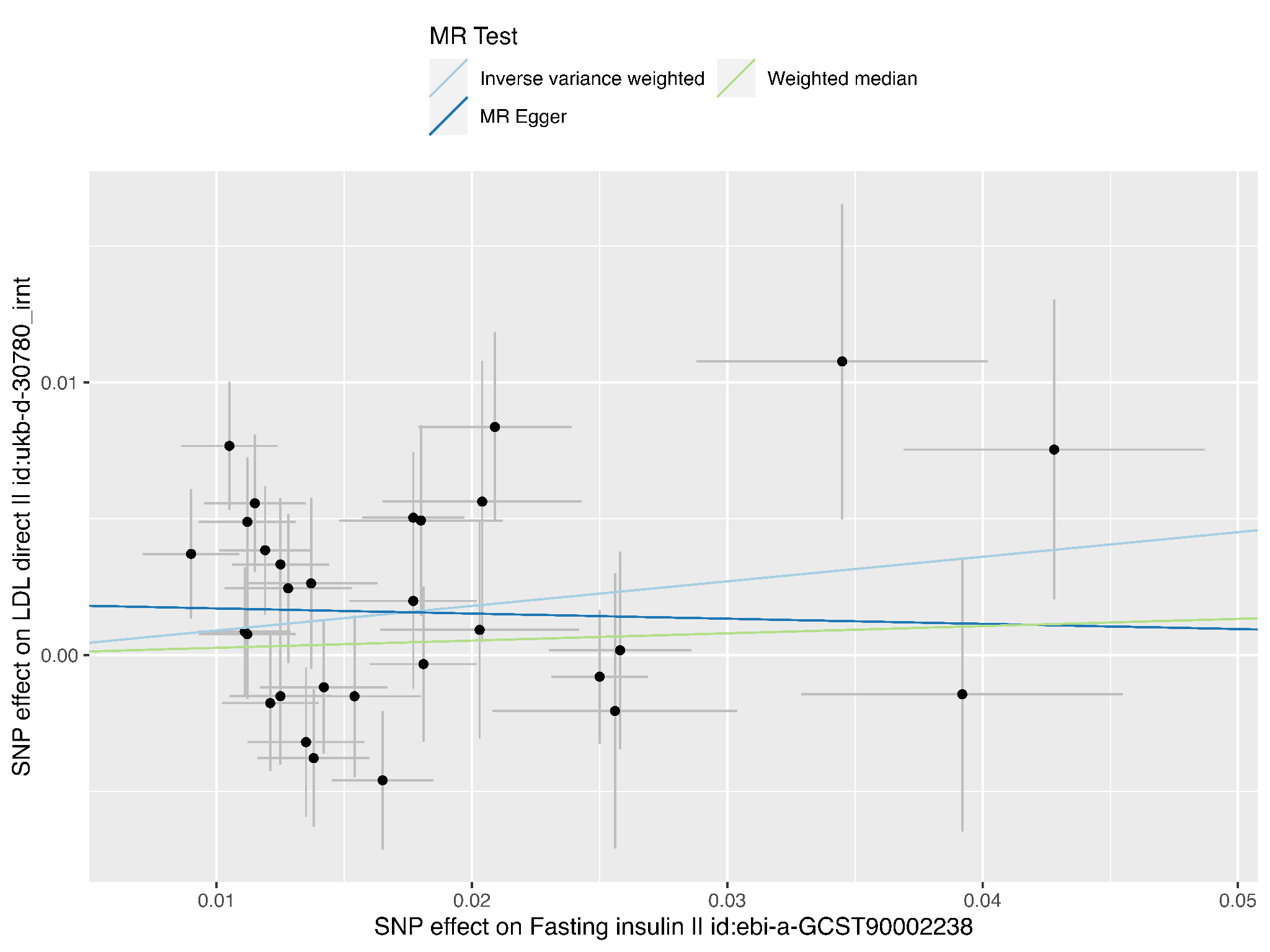


Supplementary Figure 4. Genetic associations between fasting insulin levels and LDL in liberal analysis (outlier SNP excluded)

Supplementary Figure 4 Legend. Each genetic variant included in the analysis is represented as a point + 95% CI. Localization on the horizontal axis represents the correlation of the variant with exposure (plasma fasting insulin, inverse variance normal transformed values). Localization on the vertical axis represents the correlation of the variant with the outcome (LDL-C, inverse-rank normalized). Lines represent estimates of different MR methods.


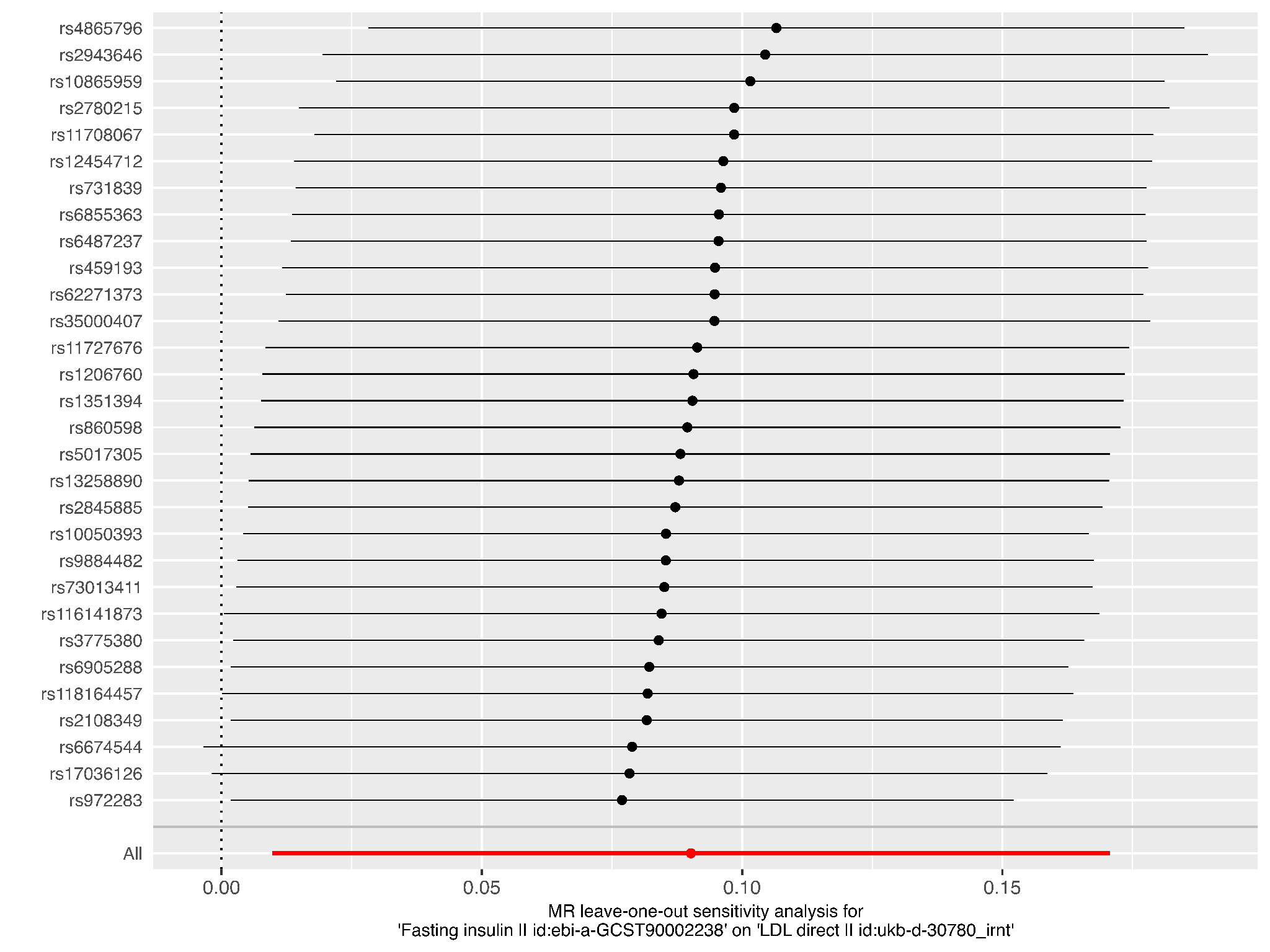


Supplementary Figure 5. Results of leave-one-out analysis

Supplementary Figure 5 Legend: Horizontal axis – inverse-variance weighted-mean estimate with 95% confidence intervals of genetic associations between insulin and LDL-C. Each dot and confidence interval represents an inverse-variance weighted mean obtained when a variant listed on the left side is removed from the analysis.
